# Cannabidiol in clinical and preclinical anxiety research. A systematic review into concentration–effect relations using the IB-de-risk tool

**DOI:** 10.1177/02698811221124792

**Published:** 2022-10-14

**Authors:** Caroline MB Kwee, Joop MA van Gerven, Fleur LP Bongaerts, Danielle C Cath, Gabriël Jacobs, Johanna MP Baas, Lucianne Groenink

**Affiliations:** 1 Department of Experimental Psychology and Helmholtz Institute, Faculty of Social and Behavioural Sciences, Utrecht University, Utrecht, The Netherlands; 2 Altrecht Academic Anxiety Centre, Utrecht, The Netherlands; 3 Centre for Human Drug Research, Leiden, The Netherlands; 4 University of Groningen, University Medical Centre Groningen, Groningen, The Netherlands; 5 Department of Specialist Trainings, GGZ Drenthe, Assen, The Netherlands; 6 Department of Pharmaceutical Sciences, Division of Pharmacology, Utrecht Institute for Pharmaceutical Sciences, Utrecht University, Utrecht, The Netherlands

**Keywords:** Cannabidiol, CBD, anxiety, anxiolytic, safety, pharmacokinetic, IB-de-risk, systematic review, translational

## Abstract

**Background:**

Preclinical research suggests that cannabidiol (CBD) may have therapeutic potential in pathological anxiety. Dosing guidelines to inform future human studies are however lacking.

**Aim:**

We aimed to predict the therapeutic window for anxiety-reducing effects of CBD in humans based on preclinical models.

**Methods:**

We conducted two systematic searches in PubMed and Embase up to August 2021, into pharmacokinetic (PK) and pharmacodynamic (PD) data of systemic CBD exposure in humans and animals, which includes anxiety-reducing and potential side effects. Risk of bias was assessed with SYRCLE’s RoB tool and Cochrane RoB 2.0. A control group was an inclusion criterion in outcome studies. In human outcome studies, randomisation was required. We excluded studies that co-administered other substances. We used the IB-de-risk tool for a translational integration of outcomes.

**Results:**

We synthesised data from 87 studies. For most observations (70.3%), CBD had no effect on anxiety outcomes. There was no identifiable relation between anxiety outcomes and drug levels across species. In all species (humans, mice, rats), anxiety-reducing effects seemed to be clustered in certain concentration ranges, which differed between species.

**Discussion:**

A straightforward dosing recommendation was not possible, given variable concentration–effect relations across species, and no consistent linear effect of CBD on anxiety reduction. Currently, these results raise questions about the broad use as a drug for anxiety. Meta-analytic studies are needed to quantitatively investigate drug efficacy, including aspects of anxiety symptomatology. Acute and (sub)chronic dosing studies with integrated PK and PD outcomes are required for substantiated dose recommendations.

## Introduction

The endocannabinoid (eCB) system is a modulator of multiple neurotransmitter systems (Kogan and Mechoulam, 2006). One of its receptors, the cannabinoid type 1 receptor (CB1R), is densely expressed throughout the brain (Herkenham et al., 1990). Consequently, cannabinoids induce a wide range of central nervous system (CNS)-mediated effects (Breivogel and Childers, 1998). Following isolation of the CB1R-binding eCBs anandamide (N-arachidonoylethanolamine (AEA)) and 2-arachidonoyl-glycerol in the 1990s (Hanuš, 2007), preclinical anxiety research has increasingly focused on the eCB system (Griebel and Holmes, 2013). Marsicano et al. (2002) showed that in mice, genetic deletion or pharmacological blockade of CB1R impaired fear extinction. Inactivation of CB1R by genetic deletion or by administration of a CB1R antagonist has also been studied with respect to its effect on unconditioned anxiety, with diverging outcomes seemingly dependent on dose, animal strain and testing conditions (Lafenêtre et al., 2007). No additional studies were performed to explain this variability in outcomes.

In humans, increased subjective anxiety has been associated with disrupted AEA signalling. For example, moderate to large negative correlations between baseline serum AEA content and anxiety levels were demonstrated in healthy volunteers (*n* = 71) (Dlugos et al., 2012) and in females with a depressive episode (*n* = 28) (Hill et al., 2008). Conversely, in a small study in unaccompanied refugee minors (*n* = 93), no significant correlations were found between hair AEA content and psychopathological symptoms (Croissant et al., 2020), and very recently negative correlations were found with plasma eCB levels and self-report anxiety scales in post-traumatic stress disorder (Leen et al., 2022).

Despite these somewhat conflicting findings, it has been argued that pharmacological inhibition of hydrolysis or reuptake of AEA, an endogenous ligand of the CB1R (Hanuš, 2007), could attenuate pathological anxiety. Bisogno et al. (2001) demonstrated that cannabidiol (CBD) inhibits AEA hydrolysis and cellular uptake of AEA. This mechanism, as well as other pharmacological activities like 5HT1A activation (Campos and Guimarães, 2008), has been related to the frequently discussed anxiety-reducing properties of this important cannabis sativa constituent (e.g. Crippa et al., 2018). In addition, CBD and AEA are agonists of the transient receptor potential vanilloid subtype 1 (TRPV1) at higher concentrations (Bisogno et al., 2001; Ross, 2003), which could be involved in the anxiogenic effects that were also reported with this compound (Campos and Guimarães, 2009). Interestingly, after initial activation of TRPV1, CBD desensitises the channel (Bisogno et al., 2001). This could, in theory, abrogate the effects of AEA at TRPV1 (Ross, 2003). However, this has yet to be experimentally confirmed.

The effects on anxiety outcomes reported in CB1R inactivation studies (Lafenêtre et al., 2007; Marsicano et al., 2002), the possible association between subjective anxiety and disrupted AEA signaling (Dlugos et al., 2012; Hill et al., 2008), and the potential of CBD to enhance levels of this endogenous ligand of the CB1R (Bisogno et al., 2001) suggest that CBD may be suitable for therapeutic use.

This is further supported by data suggesting that the compound has a favourable safety and tolerability profile. Literature reviews on human studies suggest that CBD is well tolerated up to chronic oral doses between 1500 mg (Bergamaschi et al., 2011a) and 3000 mg CBD per day (Chesney et al., 2020). The only adverse event (AE) reported to occur more frequently with CBD compared to placebo was diarrhoea (Chesney et al., 2020). In childhood epilepsy, abnormal liver function tests, pneumonia, decreased appetite, diarrhoea and somnolence occurred more frequently in CBD compared to placebo conditions (Chesney et al., 2020). These AEs could be attributed to CBD inhibiting the hepatic metabolism of other medications including anti-epileptics (Bergamaschi et al., 2011a; Chesney et al., 2020). The authors concluded that the controlled use of CBD in humans is safe, although careful monitoring for interactions with other medications is necessary (Bergamaschi et al., 2011a; Chesney et al., 2020).

Uncertainty about the effective dose range of CBD may explain the somewhat conflicting results regarding the anxiety-reducing properties of this compound. A previous narrative review by Melas et al. (2021) described anxiety reduction by CBD in anxiety tests in rodents with certain doses. Some evidence for an inverted U-shaped dose–response curve was seen in the CBD condition with the elevated plus maze (EPM) test (Melas et al., 2021). However, the dose range in which anxiety-reducing effects in the EPM test were reported varied considerably, and there were also negative results. One study in rats reported anxiety-reducing effects at 2.5, 5 and 10 mg/kg intraperitoneally (i.p.), but not at 20 mg/kg (Guimarães et al., 1990), whereas in a second study in rats, beneficial effects occurred with doses ranging from 0.5 mg/kg up to 50 mg/kg i.p. (Onaivi et al., 1990). In humans, too, an inverted U-shaped dose–response curve has been found for CBD in small samples of healthy subjects who performed a public speaking task. A dose of 300 mg orally ingested CBD elicited anxiety-reducing effects, lower and higher doses did not (Linares et al., 2019; Zuardi et al., 2017).

This limited availability of results on the relationship between dose and effect provides only an initial guideline for CBD administration in humans. The range between minimum dose for anxiety-reducing effects and maximum tolerated dose of CBD in humans is still unclear (Skelley et al., 2020). Since dosing guidelines or maximum doses for CBD are lacking (MacCallum and Russo, 2018), there is the risk of dosing too low for a therapeutic effect, which may ultimately lead to confusion about unexpected null findings. Furthermore, subjects may be exposed to undesirable drug effects that could have been avoided when knowledge about maximum tolerable exposure was available. Despite the importance of integrated assessment of preclinical and clinical dose–effect relationships of a new compound before it is administered in humans (Van Gerven and Cohen, 2018), pharmacology-based dose-selection has not been performed for CBD. This omission may at least partly be responsible for the ‘slow dawn of the long-predicted era of cannabinoid medicines’ (Young and Nutt, 2021).

The primary aim of this study was to predict the CBD plasma concentration range in which anxiety-reducing effects of CBD can be expected to occur in humans. To achieve this objective, we used the IB-de-risk tool, developed by Van Gerven and Cohen (2018). This tool summarises pharmacokinetic (PK), pharmacodynamic (PD) and safety data from an Investigator’s Brochures of novel drugs in development, but it can also be used to obtain an overview of published preclinical and clinical literature (see, e.g., Cohen et al., 2022). For the current work data on CBD doses, CBD plasma exposure levels, effects on anxiety outcomes and undesirable effects, were obtained with systematic review of the literature, and were entered in the tool. The IB-de-risk approach yields a structured, tabular and colour-coded overview from which patterns become apparent that would otherwise be very hard – if not impossible – to derive from a narrative synthesis alone. The obtained semiquantitative colour-coded overview of all the preclinical and clinical data maximises understanding of what would otherwise be separate chunks of data (Van Gerven and Cohen, 2018), and hence can aid in predicting the therapeutic window for anxiety-reducing effects of CBD in humans.

## Methods

This review was preregistered on PROSPERO (CRD42021251490 and CRD42021236572).

Protocol CRD42021236572 had already been registered with the aim of meta-analytically summarising the evidence of PD effects of anandamide breakdown and/or cellular reuptake inhibitors, including CBD. For the current review, we included only the studies in which CBD was used as a pharmaceutical, as was described in protocol CRD42021251490: ‘In order to address our overall research aim of establishing the therapeutic window of CBD in which anxiolytic effects in humans are to be expected, PK data extracted in the present review will be combined with data from a second review. This review on fear expression, fear learning and anxiety symptoms has been registered with PROSPERO (CRD42021236572)’. Hence, for the current paper, we analysed all results that concerned CBD from this broader review, as per protocol.

### Search strategy

The two systematic literature searches were conducted in line with Preferred Reporting Items for Systematic Reviews and Meta-analyses (PRISMA) guidelines. PRISMA checklists are included as Supplemental Tables 1 and 2. Studies were searched in the electronic databases PubMed and Embase using both free text and underlying terms (MeSH and Emtree, respectively) up to 19 May 2021. Only peer-reviewed studies were included. No restrictions were placed on publication year or language.

The full search strategies are found in Supplemental Table 3.

Preregistered but as of yet unpublished studies were searched as well in ClinicalTrials.gov, the EU Clinical Trials Register, the Australian and New Zealand Clinical Trials Registry, Animal Study Registry (German Centre for the Protection of Laboratory Animals) and Preclinicaltrials.eu, to get an indication of potential positive results bias.

### Inclusion and exclusion criteria

#### Studies

For human studies with anxiety outcomes, only randomised designs, in which a CBD condition was compared to a non-active placebo/vehicle condition, were eligible. The use of randomisation is usually not reported in animal research (Muhlhausler et al., 2013). Due to underreporting of this important aspect of study design, it has of yet not been empirically demonstrated whether the use of randomisation would influence outcomes. Therefore, in animals we considered vehicle-controlled experiments without information about randomisation and explicitly non-randomised but controlled studies to be eligible as well. For studies with PK outcomes, both studies with and without a control condition were considered eligible.

#### Participants

Included were studies with healthy, adult non-human mammals with a common naturally occurring phenotype, or bred or engineered for having an anxious phenotype, and with healthy adult humans or subjects diagnosed with an anxiety disorder according to the DSM criteria applied in included studies. This includes DSM-IV and DSM-5 specific phobia, social anxiety disorder (social phobia), panic disorder, agoraphobia, and generalised anxiety disorder, and DSM-IV hypochondriasis, post-traumatic stress disorder and obsessive-compulsive disorder. Experimental procedures in animals primarily aimed at inducing stress (e.g. restraint stress), rather than an anxiety(-like) response, fell beyond the scope of this paper. With regard to human studies, we excluded studies that tested chronic users of cannabis compounds; occasional use of cannabis compounds in the past was allowed, provided that subjects were in a drug-free state while participating in the experiment. Studies that allowed stable concomitant medication for anxiety and/or depression were included. Because of pregnancy-associated changes in PKs (Verstegen and Ito, 2019), studies in pregnant or lactating subjects were excluded.

#### Intervention

Studies that employed single or repeated administration of CBD were included. For within-subject designs, a washout period of at least 24 h was required to reduce carryover effects. Excluded were:

a) Experimental arms with intracerebral/intracerebroventricular/intravenous administration;
b) Experimental arms in which other substances (e.g. other cannabinoids) were co-administered as part of the investigation;
c) Experimental arms with products containing more than 0.3% Δ9-tetrahydrocannabinol on a dry weight basis;
d) For single-dose studies with anxiety outcomes, time between drug administration and anxiety assay of ⩾24 h. (For (sub)chronic dosing studies, which frequently employ ⩾24 h to distinguish delayed from acute CBD effects, time between last drug administration and anxiety assay of ⩾24 h were allowed.)

#### Outcomes

For search 1, studies were eligible for inclusion when they reported on the outcome of fear expression, fear learning (within Pavlovian fear conditioning paradigms) and/or anxiety disorder symptoms. Eligible outcome domains were subjective (humans only), neurophysiological, neuroendocrine, autonomic, behavioural and neuronal activity or connectivity during an anxiety test in brain regions involved in emotion processing and regulation. For an outcome to be eligible, outcome type had to be continuous.

For search 2, to be eligible for inclusion studies had to report on the PK outcome of *C*_max_ and/or area under the curve (AUC). In humans, absorption of CBD typically does not continue for more than 10 h after administration (even in powder form, which is associated with delayed *T*_max_) (Millar et al., 2018; Izgelov, Davidson et al., 2020a). After CBD administration via various routes in humans, *T*_max_ occurs between 0 and 5 h (Millar et al., 2018). Rats and mice treated orally with various commercially available drugs also show average *T*_max_ between 0 and 5 h (Yoshimatsu et al., 2020). To have a broad enough search window, highest reported plasma CBD levels measured within 10 h of drug administration were included as *C*_max_. Our second alternative outcome measure was the reported area under the plasma concentration curve (AUC).

### Study screening and selection

Titles and abstracts of studies retrieved using the search strategy were independently screened by the first reviewer (CK) and second reviewer (FB or one of the collaborators on the PROSPERO CRD42021236572 project) to identify studies that appeared to meet the inclusion criteria. They then independently screened the full text of these studies for eligibility. Disagreements about inclusion or exclusion were resolved through discussion, if no consensus was reached a third (LG) or fourth reviewer (JB) was consulted.

### Data extraction

All relevant data were extracted by one author (CK), 10% was extracted by a second reviewer (FB or one of the collaborators on the PROSPERO CRD42021236572 project). The results were compared, discrepancies identified and resolved through discussion (with a third reviewer (LG) and fourth reviewer (JB) when necessary). According to the population, intervention, comparison, outcome framework (Schardt et al., 2007), we recorded the details of the populations, interventions (including concomitant medication in human studies) and outcomes. The comparison group, if there was any, always received placebo/vehicle.

If PK parameters of interest (*C*_max_, AUC) were not fully reported in numbers, we requested the corresponding author to provide this information. In case no answer was received within 2 months and data were presented graphically, *C*_max_ was estimated using Plot Digitizer software (http://plotdigitizer.sourceforge.net/).

If AUC to infinity (AUC0-inf) was reported, we chose this outcome rather than AUC until the last measurable concentration (AUC0-t), provided that the dose-corrected difference between these two parameters was <20% of AUC0-t. This was used as a criterion to gauge whether the sampling interval was sufficient to adequately estimate total exposure (PhUSE CSS, 2014). If the difference was >20%, AUC0-inf was deemed inadequate and was not extracted. If AUC0-inf was not reported, we used AUC0-t, provided that the PK profile showed that plasma levels approached zero at the last measurable concentration. If this was not the case, AUC0-t was deemed inadequate and was not extracted, unless a reported elimination rate constant or elimination half-life allowed for extrapolation of AUC0-t to AUC0-inf (PhUSE CSS, 2014).

### Primary and secondary outcomes

In general, if available, we always selected results for the primary endpoint as predefined by the authors. In case of comparisons at multiple timepoints, the anxiety assessment during the anxiety test was selected as primary endpoint. If applicable (in humans), we also collected results for the assessment most closely preceding the anxiety test to assess anticipatory anxiety. Often, for the outcome of conditioned freezing (but not for other outcomes), multiple comparisons over time were reported. We then opted for the last comparison made.

We used decision rules when multiple results were available in studies. The preferred outcome measures are listed per outcome domain in Supplemental Table 4. To decide which result to collect, we established a priori, how well an outcome measure represented the outcome that it was aimed to operationalise. If the preferred outcome measures were not reported, we selected the most frequently reported outcome measure across studies employing the same anxiety test, to avoid unnecessary heterogeneity. If this outcome measure was not reported either, we selected the second most frequently reported outcome measure across studies employing the same anxiety test, etc.

For harm-related information, we searched in the included studies with the terms ‘harm’, ‘adverse’, ‘side’, ‘unwanted’, ‘undesirable’, ‘safe*’, ‘toler*’. We also included assessments of body temperature, locomotor activity and catalepsy as harm-related outcomes.

### Assessment of risk of bias

Included studies were assessed independently by two authors (CK and FB or one of the collaborators on the PROSPERO CRD42021236572 project) using the Systematic Review Centre for Laboratory animal Experimentation’s risk of bias tool for animal studies (SYRCLE’s RoB tool) (Hooijmans et al., 2014) with a vehicle control group. We used version 2 of the Cochrane risk of bias tool (RoB 2.0) (Sterne et al., 2019) for human outcome studies.

The following types of bias were assessed for the review of anxiety outcomes (terms corresponding to the SYRCLE’s RoB tool and Cochrane RoB 2 tool, respectively):

For animal studies:

(1) Sequence generation; (2) Baseline characteristics; (3) Allocation concealment; (4) Random housing; (5) Blinding of caregivers and investigators; (6) Random outcome assessment; (7) Blinding of outcome assessor; (8) Incomplete outcome data; (9) Selective outcome reporting and (10) Other (conflicting interests).

For human studies:

(1) Bias arising from the randomisation process; (2) Bias due to deviations from intended interventions; (3) Bias due to missing outcome data; (4) Bias in measurement of the outcome and (5) Bias in selection of the reported result.

Since existing tools are aimed at effect studies, and are not applicable to studies with PK outcomes, we assessed bias for the PK review by considering the following:

1. Bias due to confounders

Was food intake prior to dosing reported? Was, in human studies, concomitant medication reported? Were, in human studies, drugs-of-abuse tests conducted? Was dissolving vehicle reported when drugs were administered orally?

2. Bias due to missing data

Were outcome data available for all, or nearly all, participants? Were reasons for missing data reported and if yes, is it likely that results were biased because of missing data? Is there evidence that results were robust to the presence of missing data?

3. Bias in measurement of the outcome

Were analytical quality control and method validation procedures reported?

To assess bias in studies with harm-related objectives, we considered the following (based on the CONSORT extension on reporting of HARMS) (Ioannidis et al., 2004):

1. To what extent were study subjects aware of the potential AEs associated with the substance they were taking?
2. Was collection and assessment of safety information blinded?
3. Was the manner in which safety information was collected described clearly and thoroughly?
4. Was it clearly reported which AEs/safety outcomes occurred in which treatment arm?
5. Were discontinuations/withdrawals due to safety-related events clearly reported?
6. Was it clear up until when participants safety information was collected?

The Rob 2.0 tool has the option for judging each type of bias as ‘high’. In addition, the development group for Cochrane RoB 2.0 recommends that a result should be judged as high risk of bias when some concerns exist for multiple types of bias at the same time (Higgins et al., 2021). For the other risk assessments, we opted for the term ‘unclear’ rather than ‘some concerns’ or ‘high concerns’, because these assessments do not have a strong empirical basis (Ioannidis et al., 2004; Vollert et al., 2020).

### Data synthesis

#### Outcome categorisation

Comparisons between CBD and placebo/vehicle arm(s) as mentioned by the authors in the studies were used to decide on the presence/absence and direction of CBD treatment effects on fear learning, fear expression and anxiety symptoms. If authors did not explicitly report statistical significance for a CBD versus vehicle/placebo comparison (Todd and Arnold, 2016), we interpreted this as a non-significant difference.

If multiple outcomes belonging to more than one outcome domain (see Supplemental Table 4) were used within an experiment, we considered these as one observation. If the authors reported a significant result on at least one of these outcomes, the observation was categorised as representing a significant CBD effect.

Harm-related hypotheses and hypothesis tests are uncommon (Ioannidis et al., 2004). Nevertheless, for CBD we expected CNS inhibition (Rosenkrantz et al., 1981), which could lead, for instance, to decreased motor activity, sedation or somnolence. Next to type of AE, we gauged relatedness of AE to CBD by comparing AE occurrence between CBD and placebo/vehicle conditions. A higher frequency in the CBD condition would argue for relatedness to this compound. Also, a dose–response relationship in the form of increasing occurrence of an AE with increasing doses of CBD increases the probability of CBD relatedness.

We categorised information on harms based on clinical severity. We based our categorisation on The Veterinary Cooperative Oncology Group – Common Terminology Criteria for Adverse Events (LeBlanc et al., 2016). Our categories were ‘AE very mild/infrequent and/or uncertain relationship to CBD’, ‘undesirable effects’ (mild or moderate clinical signs, self-limiting, not requiring intervention, or non-invasive intervention indicated, relatedness to CBD probable), ‘more severe AEs’ (medically significant but not immediately life threatening, relatedness to CBD probable), ‘serious irreversible toxicity and/or death’.

A colour-coded overview of the outcomes was construed using the IB-de-risk tool (Van Gerven and Cohen, 2018), which contains all the studies included in the data synthesis. Each row contains a separate observation. First, experiments within studies were considered as separate observations. Second, studies in which different doses were used within an experiment were considered as separate observations for each administration. Third, measurements of anxiety outcomes and of potential side effects were considered as separate observations. Rows were sorted by *C*_max_ and AUC, measured or otherwise imputed, to obtain an impression of a concentration–effect association. The colour coding scheme, which was based on outcome categorisation, is shown in Table 1.

**Table 1.** Colour coding scheme used for the overview of the outcomes.

White	No anxiety outcomes measured
Light green	Anxiety outcomes measured, no effect observed
Green	Anxiolytic effects
Light yellow	AE very mild/infrequent and/or uncertain relationship to CBD
Yellow	Undesirable effects
Orange	More severe adverse effects
Red	Serious irreversible toxicity and/or death
Pink	Imputed PK parameter
Grey	No PK estimation could be made

AE: adverse event; CBD: cannabidiol; PK: pharmacokinetic.

#### Imputation of PK parameters

To estimate the relation between systemic exposure and therapeutic or undesirable effects across different species and studies, we inferred maximum plasma concentration (*C*_max_) and AUC for studies that measured anxiety outcomes, but did not include this PK information. We did so by using papers that reported CBD's PK parameters of systemic exposure in the same species.

Results from an earlier review suggest that the use of lipid formulations and subjects being in a fed state increases *C*_max_ and AUC (Millar et al., 2018). We therefore matched PK studies and experiments that focused on anxiety outcomes on these parameters, before estimating missing PK parameters. We used linear inter- or extrapolation (per administration mode, per species) for our estimations. Non-linear trendlines were fit when visual inspection of plots suggested a non-linear association. We subsequently selected the method with the largest explained variance. Rows were then sorted by *C*_max_ or AUC (measured or otherwise imputed).

With multiple dosing, accumulation of CBD in human adipose tissues leads to prolonged elimination half-life (Lucas et al., 2018) up to around 68 h with multiple dosing (Hosseini et al., 2020; Taylor et al., 2018). Therefore, even with administration once daily, CBD is eliminated incompletely from the body at the time a new dose is given. Dose-dependent moderate drug accumulation was reported at steady state (1.8- to 2.6-fold for 750 and 1500 mg bidaily doses) (Hosseini et al., 2020; Taylor et al., 2018). This indicates that PK estimates for multiple dose studies would require complex PK modelling. Since we consider this to be beyond the scope of this paper, we limited ourselves to estimating missing plasma exposure levels only for single-dose studies.

Details about PK estimates for single-dose human and animal studies are described in the Supplemental Material.

#### Interpretation

We provided a narrative synthesis of the findings discussing between-species translatability, anticipated effective human dose and safety margin using the colour-coded overview. More specifically, we inspected our colour-coded overview for the presence/absence of different levels of severity of AEs, and the drug concentrations with which these AEs occurred. The lowest drug concentrations with predominantly ‘desirable effects’ constitute the lower level of the therapeutic range.

### Risk of bias due to missing results in the synthesis

To assess selective reporting bias, we compared the tests and outcomes planned by the original investigators with those reported in the published study. When published protocols were not available, we compared the methods and the results sections.

## Results

### Results of searches

**Figure 1.**
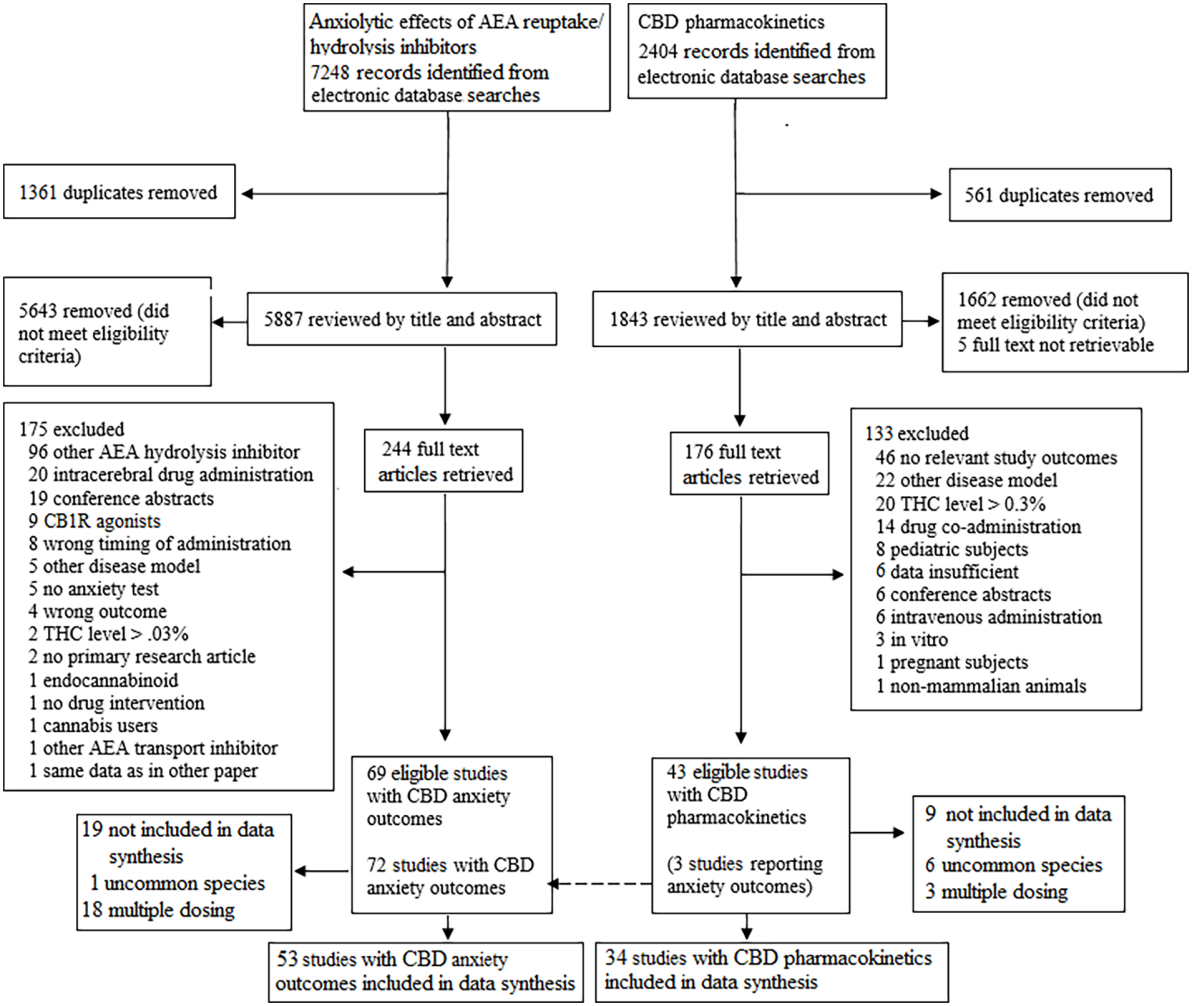
Flowchart displaying the study selection process. AEA: anandamide; CBD: cannabidiol; CB1R: cannabinoid type 1 receptor; THC: Δ9-tetrahydrocannabinol.

With our PD search that was focused on anxiety outcomes, we found 7248 records. After duplicates removal, we screened 5887 records, from which we reviewed 244 full-text articles and included 69 studies. Of these studies, 53 were included in the data synthesis. With our PK search that focused on PK outcomes, we found 2404 records. After duplicates removal, we screened 1843 records, from which we reviewed 176 full-text articles and included 43 studies. Of these studies, 34 were included in the data synthesis. The selection processes for both searches are displayed in Figure 1. Ongoing and incomplete studies are displayed in Supplemental Table 5.

### Description of studies included in the data synthesis

The included studies and their characteristics are presented in Supplemental Table 6 (studies with anxiety outcomes) and Supplemental Table 7 (studies with PK data).

In all human studies, the administration route was oral (p.o.). In mice and rats, drugs were predominantly administered via the i.p. route. Across studies, the most frequently assessed outcome domain was behavioural and the most frequently used anxiety test the EPM.

In humans, nine studies reported anxiety outcomes (Bergamaschi et al., 2011b; Crippa et al., 2004, 2011, 2021; Fusar-Poli et al., 2009, 2010; Linares et al., 2019; Zuardi et al., 1993, 2017) and 14 studies reported PK outcomes but no anxiety outcomes (Atsmon et al., 2018; Crockett et al. 2020; Grimm et al., 2018; Hosseini et al., 2020; Knaub et al., 2019; Martín-Santos et al., 2012; Patrician et al., 2019; Perkins et al., 2020; Schoedel et al., 2018; Taylor et al., 2018, 2019; Tayo et al., 2020; Verrico et al., 2020; Williams et al., 2021). In three studies, both types of outcomes were reported (Crippa et al., 2021; Fusar-Poli et al., 2009, 2010).

In mice, 20 studies reported anxiety outcomes (Aso et al., 2019; Assareh et al., 2020; Breuer et al., 2016; Casarotto et al., 2010; Deiana et al., 2012; Florensa-Zanuy et al., 2021; Kasten et al., 2019; Long et al., 2010, 2012; Myers et al., 2019; Nardo et al., 2014; Navarrete et al., 2018; Onaivi et al., 1990; Schiavon et al., 2016; Todd and Arnold, 2016; Todd et al., 2017; Twardowschy et al., 2013; Uribe-Mariño et al., 2012; Zagzoog et al., 2020; Zieba et al., 2019) and four studies reported PK outcomes but no anxiety outcomes, or no CBD-vehicle comparison was reported (Anderson et al., 2021; Brzozowska et al., 2016; Majimbi et al., 2021; Pang et al., 2021). In two studies, both types of outcomes were reported (Deiana et al., 2012; Zieba et al., 2019).

In rats, 24 studies reported anxiety outcomes (Almeida et al., 2013; Espejo-Porras et al., 2013; Gáll et al., 2020; Gazarini et al., 2014; Guimarães et al., 1994; Hložek et al., 2017; Javadi-Paydar et al., 2019; Jurkus et al., 2016; Kajero et al., 2020; Karniol et al., 1974; Lemos et al., 2010; Mahmud et al., 2017; Malone et al., 2009; Martín-González et al., 2018; Moreira et al., 2006; Murkar et al., 2019; O’Brien et al., 2013; Resstel et al., 2006; Rock et al., 2017; Shallcross et al., 2019; Shoval et al., 2016; Song et al., 2016; Stern et al., 2012, 2015) and 10 studies reported only PK outcomes (Cherniakov et al., 2017; Deiana et al., 2012; Feng et al., 2021; Izgelov et al., 2020b, 2020c, 2020d; Nagao et al., 2020; Paudel et al., 2010; Xu et al., 2019; Zgair et al., 2016). In one study, both types of outcomes were reported (Hložek et al., 2017).

As shown in Figure 1, 28 studies (*n* = 19 with anxiety outcome, *n* = 9 with CBD PKs) that were initially eligible for this review, were not included in the data synthesis.

First, multiple dose regimens were not included because of the complex PK modelling that would be required which would not be conceivable with the available data. For this reason, one study with anxiety outcomes (Masataka, 2019) and one with PK outcomes in humans (Taylor et al., 2020) were not included in the synthesis. Two studies with multiple dosing and PK objectives (Bartner et al., 2018; Vaughn et al., 2020) and one with anxiety outcomes in dogs (Morris et al., 2020) were not included. For the same reason, the following studies with PD outcomes in mice and rats were not included: Bis-Humbert et al. (2020), Campos et al. (2012, 2013); Cheng et al. (2014a, 2014b), Coles et al. (2020), Elbatsh et al. (2012), Fogaça et al. (2018), Luján et al. (2018, 2020), Murphy et al. (2017), Pang et al. (2021), Schleicher et al. (2019), Silvestri et al. (2020), Watt et al. (2020a, 2020b).

Second, the following studies that contained PK data for cats (Kulpa et al., 2021), horses (Ryan et al., 2021), minipigs (Wray et al., 2017), guinea pigs (Paudel et al., 2010) and rabbits (Mannila et al., 2007), and PD data from one study which assessed CBD effects on startle in capuchin monkeys (Saletti et al., 2017) were excluded. We could either not use the PK data to estimate plasma exposure levels in similar species (Mannila et al., 2007; Paudel et al., 2010; Wray et al., 2017) or PK data were not available to estimate plasma exposure levels (Saletti et al., 2017). Third, PK and PD results for cats (Kulpa et al., 2021) and horses (Ryan et al., 2021) were not included in the synthesis. These species are uncommonly used as a model to predict human kinetics and toxicity; the translational value may be limited (The National Institute of Public Health and the Environment and 3Rs-Centre Utrecht Life Sciences, 2015).

### Risk of bias of studies included in the data synthesis

We analysed risk of bias per study, given the overlap of aspects that could lead to bias between experiments in the same studies. A summary is provided in Supplemental Figure 5.

Overall risk of bias was unclear for anxiety outcomes in all animal studies due to lack of information about blinding, dropout and/or handling of missing data, and randomisation. A high degree of similarity between CBD and control condition could often be assumed, since animals were housed under controlled conditions, were almost invariably of the same sex (male), and often, animals were habituated to the testing environment before submission to the anxiety test.

Our overall risk of bias judgements for human studies with respect to anxiety outcomes ranged from low to high. All human studies were randomised and used identical appearing capsules to conceal the allocation to CBD and placebo treatments. In general, risk of bias due to missing outcome data was considered low, as in most studies, numbers of patients after randomisation were equal to the number of patients for whom results were available (Bergamaschi et al., 2011b; Crippa et al., 2004, 2011; Linares et al., 2019). In contrast, highly variable CBD plasma concentrations (*M* = 17; standard deviation = 29 ng/mL) (Fusar-Poli et al., 2009, 2010) may have led to biased estimates of per-protocol effects. Furthermore, increased mental sedation in the CBD condition may have affected subjective anxiety ratings (Crippa et al., 2004).

Most human studies used healthy volunteers and described restrictions concerning the use of recreational drugs. However, concomitant medication use and drugs-of-abuse tests were not reported in several papers (Bergamaschi et al., 2011b; Crippa et al., 2004, 2011, 2021; Crockett et al., 2020; Hosseini et al., 2020; Izgelov et al., 2020a; Knaub et al., 2019; Linares et al., 2019; Patrician et al., 2019; Schoedel et al., 2018; Taylor et al., 2018, 2019; Tayo et al., 2020; Williams et al., 2021; Zuardi et al., 1993). Information about bioanalytical methods validation in studies with PK data varied from no description (e.g. Izgelov et al., 2020a) to a detailed one (e.g. Perkins et al., 2020). For the majority of PK studies, overall risk of bias was unclear.

For animal studies with harm-related outcomes but without explicit harm-related study objectives, overall risk of bias was unclear due to underreporting of information needed to assess bias. For all human studies, it was unclear whether participants knew beforehand whether information on harms was collected, and whether assessment of safety information was blinded. Method of assessing AEs was usually described, although sometimes concise. Period of assessing safety was usually specified, but relatively short, with some exceptions (e.g. a follow-up period of 8–14 days (Schoedel et al., 2018) and 2 weeks (Taylor et al., 2019).

### PD effects across species

PD results are summarised using the colour coding scheme in Table 1. As shown in Supplemental Table 8, no clear pattern of associations between *C*_max_, AUC and frequency of anxiety-reducing or adverse effects were discernible, when looking at all species and anxiety tests together. Across species, for the majority of observations, CBD had no effect on anxiety outcomes (121 out of 172; 70.3%). Importantly, 138 of 172 rows (80.2%) were observations from studies that investigated multiple doses of CBD without necessarily expecting an anxiety-reducing effect with each dose. Anxiety-reducing effects were reported across the entire range of systemic exposure (300–53,000 ng/mL × h). Regardless of effect on anxiety outcome, sample sizes per experimental condition were rather small (between *n* = 5 and *n* = 22). There were in total 19 rows with very mild AEs, infrequent AEs and/or AEs with an uncertain relationship to CBD.A comparable number of rows (*n* = 22) contained observations of mild or moderate clinical signs that were probably related to CBD. The absence of severe AEs (which would be coloured orange or red) with plasma levels that are adequate to measure anxiety-reducing effects (indicated by the colour green) is in line with the advantageous safety profile of CBD in humans reported in earlier work (Bergamaschi et al., 2011a; Chesney et al., 2020).

### PD effects within species

In the paragraphs below, we describe the most active AUC range per species, that is, the range in which anxiety-reducing effects of CBD occurred relatively frequently, when compared to other AUC levels. Sorting the data on *C*_max_ did not change the pattern of results that was obtained by sorting on AUC.

In humans, we identified the AUC range (Table 2) with the most frequent observations of anxiety-reducing effects, to be between ~2000 and 2800 ng/mL × h. In this most active AUC range, there were four of seven rows with anxiety-reducing effects with 300–600 mg CBD doses (Bergamaschi et al., 2011b; Crippa et al., 2004, 2011; Linares et al., 2019). Within the most active range, 2 of 12 effects were consistent with CNS inhibition that could be related to CBD: Increased sedation in the CBD condition compared to placebo (Bergamaschi et al., 2011b; Crippa et al., 2004). Only one study was conducted that measured anxiety outcomes with higher total systemic exposure (~3700 ng/mL × h). This study reported no anxiety-reducing effect of CBD (Zuardi et al., 2017).

**Table 2.** Colour-coded overview of the most active AUC range in humans.

Study ID	Type of anxiety test	Dose (mg)	Effects		AUC (ng/mL × h)	Classification (see legend)
Linares et al. (2019)	SPS	300	Anxiety-reducing effect on VAMS during speech	Low AUC	2003	
Zuardi et al. (1993)	SPS	300	No effect	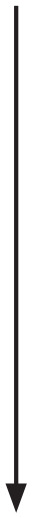	2003	
Zuardi et al. (2017)	SPS	300	No effect		2003	
Crippa et al. (2004)	fnct	400	Lower VAMS anxiety at 75 min, modulated ECD uptake		2277	
Crippa et al. (2004)	fnct	400	Higher mental sedation at 75 min		2277	
Crippa et al. (2011)	fnct	400	Lower VAMS anxiety at 75 min, modulated ECD uptake		2277	
Bergamaschi et al. (2011b)	SPS	600	Lower VAMS anxiety during speech		2827	
Bergamaschi et al. (2011b)	SPS	600	Less decrease in sedation anticipating speech	High AUC	2827	
Linares et al. (2019)	SPS	600	No effect		2827	

Effects are displayed in brief, an overview of all rows and more elaborate results is found in Supplemental Table 8.

AUC: area under the curve; ECD: ethyl cysteinate dimer; fnct: functional neuronal activation; green: anxiety-reducing effects; light green: anxiety outcomes measured, no effect observed; pink: imputed PK parameter; SPS: simulated public speaking; VAMS: Visual Analogue Mood Scale; yellow: undesirable effects.

In rats, sorting rows on AUC yielded the most active range between ~1500 and 2900 ng/mL × h (Table 3), with 16 of 26 anxiety-reducing effects (Gazarini et al., 2014; Guimarães et al., 1990, 1994; Lemos et al., 2010; Mahmud et al., 2017; Martín-Gonzáles et al., 2018; Moreira et al., 2006; Resstel et al., 2006; Rock et al., 2017; Shallcross et al., 2019; Song et al., 2016; Stern et al., 2012) and two effects that may be interpreted as anxiety increasing (Gáll et al., 2020; Song et al., 2016). As shown in Supplemental Table 8, null effects became more frequent with lower AUC; between ~300 and 1100 ng/mL × h there were only 3 of 15 anxiety-reducing effects. One study reported increased motor activity after CBD administration, with AUC of ~500 ng/mL × h (Hložek et al., 2017). Similarly, with larger AUC; between ~4400 and 17,700 ng/mL × h, there were only 4 of 22 anxiety-reducing effects (Murkar et al., 2019; Shoval et al., 2016; Stern et al., 2012). There were three cases of CBD effects on vertical and horizontal activities (Espejo-Porras et al., 2013). Drowsiness and piloerection in rats, which were categorised as severe AEs, occurred after a single dose at the high end of the AUC range (>40,000 ng/mL × h; Deiana et al., 2012).

**Table 3.** Colour-coded overview of the most active AUC range in rats.

Study ID	Type of anxiety test	Dose (mg/kg)	HED (mg) (×60 kg)	Effects		AUC (ng/mL × h)	Classification (see legend)
Almeida et al. (2013)	SI	5	1440	No effect	Low AUC	1473	
Guimarães et al. (1990)	AA	5	1440	Higher % open arm entries	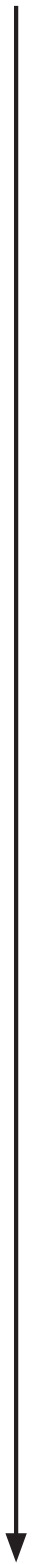	1473	
Guimarães et al. (1994)	AA	5	1440	Higher % open arm entries		1473	
Jurkus et al. (2016)	FC d	5	1440	No effect		1473	
Malone et al. (2009)	SI	5	1440	No effect		1473	
Rock et al. (2017)	AA	5	1440	No effect		1473	
Rock et al. (2017)	S_AA	5	1440	Higher mean time in light box		1473	
Shallcross et al. (2019)	AA	5	1440	Decreased latency to enter the light compartment		1473	
Moreira et al. (2006)	FC d	5	1440	No effect		1473	
Moreira et al. (2006)	FC d	5	1440	No effect		1473	
Gáll et al. (2020)	AA	10	2880	Less rearing compared to vehicle		2945	
Lemos et al. (2010)	fnct	10	2880	Attenuation of c-Fos expression in BNST after conditioning		2945	
Mahmud et al. (2017)	AA	10	2880	More time in open arms than vehicle		2945	
Moreira et al. (2006)	FC d	10	2880	Higher no. of punished licks		2945	
Moreira et al. (2006)	FC d	10	2880	Higher no. of punished licks		2945	
Resstel et al. (2006)	FC	10	2880	Lower % time freezing, less increase in heart rate		2945	
Song et al. (2016)	FC	10	2880	Lower % freezing time at test		2945	
Song et al. (2016)	FC	10	2880	Increased % freezing time at test		2945	
Stern et al. (2012)	FC	10	2880	Lower % freezing time during context re-exposure		2945	
Stern et al. (2012)	FC	10	2880	Lower % freezing time during context re-exposure		2945	
Stern et al. (2012)	FC	10	2880	Lower % freezing time during context re-exposure		2945	
Karniol and Carlini (1974)	AA	10	2880	No effect		2945	
Lemos et al. (2010)	FC	10	2880	Lower % freezing during context re-exposure		2945	
Gáll et al. (2020)	AA	10	2880	No effect		2945	
Gáll et al. (2020)	AA	10	2880	No effect		2945	
Gazarini et al. (2014)	FC	10	2880	Lower % freezing during context test		2945	
Guimarães et al. (1990)	AA	10	2880	Higher % open arm entries	High AUC	2945	
Jurkus et al. (2016)	FC d	10	2880	No effect		2945	

Effects are displayed in brief, an overview of all rows and more elaborate results is in Supplemental Table 8.

AA: approach avoidance; AUC: area under the curve; FC: fear conditioning to context; FC d: fear conditioning to discrete cue; fnct: functional neuronal activation; green: anxiety-reducing effects; light green: anxiety outcomes measured, no effect observed; pink: imputed PK parameter; S: exposed to stressor(s); SI: social interaction; yellow: undesirable effects.

In mice, the most active range (Table 4) seemed to be between ~10,500 and 13,300 ng/mL × h, with 11 of 17 anxiety-reducing effects (Assareh et al., 2020; Breuer et al., 2016; Casarotto et al., 2010; Nardo et al., 2014; Uribe-Mariño et al., 2012). With lower AUC, between ~4400 and 8800 ng/mL × h, there were 4 of 34 rows with an anxiety-reducing effect (Deiana et al., 2012; Todd and Arnold, 2016; Todd et al., 2017; Zieba et al., 2019) and 1 of 34 rows with an anxiety-increasing effect (Kasten et al., 2019). With higher AUC, between ~22,100 and 53,000 ng/mL × h, (1/9) of results were anxiety reducing (Deiana et al., 2012) and 1 of 9 anxiety increasing (Long et al., 2012). Mice seemed less sensitive to CBD compared to humans and rats. Within the most active AUC range in humans and rats, between ~1700 and 2200 ng/mL × h, only 2 of 13 effects in mice were anxiety reducing (Kasten et al., 2019; Schiavon et al., 2016; Twardowschy et al., 2013; Uribe-Mariño et al., 2012). There were no publications of undesirable effects in mice other than the above-mentioned anxiety-increasing effects (Kasten et al., 2019; Long et al., 2012).

**Table 4.** Colour-coded overview of the most active AUC range in mice.

Study ID	Type of anxiety test	Dose (mg/kg)	HED (mg) (×60 kg)	Effects		AUC (ng/mL × h)	Classification (see legend)
Breuer et al. (2016)	RCLB	15	72	No effect	Low AUC	10,458	
Casarotto et al. (2010)	RCLB	15	72	Reduced marble burying	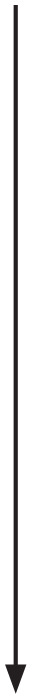	10,458	
Assareh et al. (2020)	FC d	30	144	Lower % freezing		13,254	
Assareh et al. (2020)	FC	30	144	No effect		13,254	
Assareh et al. (2020)	S_AA	30	144	No effect		13,254	
Breuer et al. (2016)	RCLB	30	144	Reduced marble burying		13,254	
Breuer et al. (2016)	RCLB	30	144	Reduced marble burying		13,254	
Breuer et al. (2016)	RCLB	30	144	Reduced marble burying		13,254	
Florensa-Zanuy et al. (2021)	AA	30	144	No effect		13,254	
Nardo et al. (2014)	RCLB	30	144	Reduced marble burying		13,254	
Nardo et al. (2014)	AA	30	144	No effect		13,254	
Schiavon et al. (2016)	AA	30	144	No effect		13,254	
Uribe-Mariño et al. (2012)	Defence	30	144	Lower behavioural index for defensive immobility outside		13,254	
Casarotto et al. (2010)	RCLB	30	144	Reduced marble burying		13,254	
Casarotto et al. (2010)	RCLB	30	144	Reduced marble burying		13,254	
Casarotto et al. (2010)	RCLB	30	144	Reduced marble burying	High AUC	13,254	
Casarotto et al. (2010)	RCLB	30	144	Reduced marble burying		13,254	

Effects are displayed in brief, an overview of all rows and more elaborate results is found in Supplemental Table 8.

AA: approach avoidance; AUC: area under the curve; FC: fear conditioning to context; FC d: Fc to discrete cue; green: anxiety-reducing effects; light green: anxiety outcomes measured, no effect observed; pink: imputed PK parameter; RCLB: repetitive compulsive-like behaviour; S: exposed to stressor(s); yellow: undesirable effects.

In summary, our synthesis revealed a predominance of null effects on anxiety outcomes in all investigated species. Yet, for all species, the colour-coded patterns may suggest an AUC range with a relatively high number of anxiety-reducing effects. For mice, the anxiety-reducing effects were predominantly observed in the marble burying test of repetitive, compulsive-like behaviour. Therefore, it is not possible to differentiate between a contribution of type of anxiety test and level of CBD exposure to these anxiety-reducing effects.

### Risk of bias due to missing results in the synthesis

Overall, there were some concerns about risk of bias due to missing results in the synthesis.

Four preregistered studies that were completed have not yet published their results (NCT03164512, NCT04577612, NCT04790136, ACTRN12620000891921), which may be indicative of positive results bias. Within published studies, anxiety tests and outcomes described in the methods sections generally matched those reported in the results sections. However, in EPM tests, authors sometimes reported one, but not both of the conventional indices of anxiety (% open arm entries and open arm time) (Rodgers et al., 1997). This may be indicative of outcome reporting bias. In addition, more extensive reporting of an animals behavioural repertoire was rare. Safety assessments were often described in a concise way in methods and results sections, which led to an unclear risk of bias due to selective reporting.

Our synthesis was limited to single-dose regimens; multiple dose regimens were not included because the required complex PK modelling, combined with the sparsity of PK data after multiple dosing. While it is unknown how the plasma levels with multiple dose regimens relate to those with single dose regimens, the majority of effects with multiple dose regimens (93 out of 114; 81.6%) constituted no differences between CBD and placebo on anxiety outcomes.

## Discussion

Preclinical research suggests that CBD may have beneficial effects in the treatment of pathological anxiety. To inform future human studies, the purpose of this study was a translational prediction of the exposure range for anxiety-reducing effects of CBD, based on its minimum exposure for anxiety-reducing effects and maximum tolerated exposure. We used the IB-de-risk tool (Van Gerven and Cohen, 2018) to synthesise PK and PD data of systemic CBD exposure in humans and animals.

Our data synthesis did not show straightforward dose–response relationships, between systemic exposure and anxiety-reducing effects, which would be expected for typical pharmacologically active drugs (Van Gerven and Cohen, 2018). None of the species showed a dose-related transition from a no-effect range, through a therapeutic anxiety-reducing range, to increasingly frequent and severe adverse effects. Across species, anxiety-reducing effects were reported within an exorbitant range of CBD exposures (~300 to 53,000 ng/mL × h). Within this range of systemic exposure, a majority of studies in our review reported no anxiety-reducing effects of CBD. Furthermore, mild to moderate AEs were observed with the same levels of drug exposure that produced anxiety-reducing effects, and the intensity of adverse effects did not increase clearly with dose. Within species, concentration ranges were discernible in which anxiety-reducing effects of CBD occurred relatively frequent. Importantly, even in these ranges, anxiety-reducing effects were interspersed with null effects.

These findings seem to be in contrast with the therapeutic potential in treating anxiety symptoms which has been described by other authors (e.g. Crippa et al., 2018) and might be an explanation for the ‘slow dawn of the long-predicted era of cannabinoid medicines’ (Young and Nutt, 2021). Although this review showed exposure–response relationships that are poorly translational and far from conventional, it would be premature to conclude that CBD does not have anxiety-reducing properties. Several alternative explanations for the lack of a clear cross-species concentration–effect relation are conceivable. Up until the beginning of the 21st century, the scientific literature contained fewer null findings than nowadays, because these findings were less likely to be to published (Shrout and Rodgers, 2018). Our comprehensive systematic literature searches may have yielded a more or less balanced representation of the literature. This includes studies with null findings, which may be attributed to individual study characteristics.

First, anxiety tests typically tap into only certain aspects of anxiety symptomatology (Sams-Dodd, 2006). It is conceivable that potentially beneficial effects of CBD are limited to some symptom dimensions of anxiety. Moreover, some anxiety tests are poor models of an anxiety disorder or anxiety symptoms, and suitability to measure anxiety-reducing drug effects may differ greatly between these tests (Bach, 2022). However, the strength of the IB-de-risk tool is to summarise all effects, without cherry-picking, to allow an overall perspective.

Beneficial effects of CBD might also be specific for anxious sub-populations with specific biological features. For example, sex- and brain region-specific differences in CB1R density in mice were induced by early life stress (Dow-Edwards, 2020) and sub-chronic stress during adult life (Zoppi et al., 2011). Furthermore, in healthy humans, changes in eCB plasma levels in response to acute stress were larger in men than in women (Dlugos et al., 2012). Behavioural effects of exogenous cannabinoids may be more or less pronounced dependent on such differences in the eCB system (Martín-Sanchez et al., 2021).

A third explanation for null effects may be the less than optimal timing between drug administration and anxiety tests to measure therapeutic effects in some studies. Levels of CBD in the brain may continue to rise after peak concentration in plasma (Deiana et al., 2012). While the former is of primary interest considering expected CNS-related effects, estimations of the latter, are commonly being used as benchmark for test commencement. In addition, after oral administration, the time at which plasma levels are highest may differ substantially between individual subjects. This has been demonstrated for rats (Cherniakov et al., 2017: 27) and humans (Taylor et al., 2018: 1061), but to a lesser extent for mice; Pang et al. (2021: 2044) reported no differences in *T*_max_ between six mice. In addition, *T*_max_ strongly depends on the formulation used in rats (Cherniakov et al., 2017: 27), mice (Majimbi et al., 2021: 8) and humans (Izgelov, Davidson et al., 2020a: 3). Thus, in some cases, the anxiety test may have already been terminated at the time plasma levels of CBD have reached their peak.

Lastly, included studies may have been underpowered to detect modest CBD effects, because of the generally small samples sizes used. A future meta-analysis may be helpful to qualitatively summarise the findings across studies while taking imprecision of reported effects into account. Moreover, such an endeavour may help to elucidate whether the effect of CBD on anxiety outcomes is dependent on certain study characteristics, such as the specific anxiety features that are under investigation, and the corresponding anxiety-related tests.

As stated above, there was no evidence of a clear exposure- or concentration–effect association across species. The lack thereof could at least partly be attributed to differences in active ranges between species. That is, rats and humans seemed more sensitive to CBD effects than mice. In rats and humans, beneficial CBD effects on anxiety outcomes were clustered in a range of concentrations around ~2000 ng/mL × h. In humans, this corresponds to oral dosages between 300 and 600 mg. Studies using higher dosages are largely lacking in humans, but in rats null effects became again more frequent with higher concentrations. In mice, the same pattern as in rats was observed in the order of fivefold increased concentrations. Anxiety-reducing effects clustered at moderate plasma concentrations (~11,000 ng/mL × h) and more numerous null effects occurred at higher plasma concentrations. It has been suggested that CBD exhibits a complex inverted U-shaped exposure–response relationship (Zuardi et al., 2017).

At present, there is no agreed upon explanation for why anxiety-reducing effects would disappear with higher concentrations. There are various explanations for such patterns (Calabrese & Baldwin, 2001). One possibility is that therapeutic activity is overcome by adverse effects at higher doses or concentrations. This review does not provide arguments for this explanation, because none of the species showed a clear increase of adverse effects with higher CBD levels. It has also been suggested that biphasic effects of CBD could be attributed to its multiple, partly antagonistic receptor targets that may be activated at different concentrations. This could, for example, involve the activation of the TRPV1 by CBD at higher concentrations (Campos and Guimarães, 2009). Data from Campos and Guimarães (2009) lend support to this notion. That is, the anxiety-reducing effect of CBD in the EPM test in lower doses disappeared with increasing doses, but was rescued by coadministration of a TRPV1 antagonist.

This is the first study to synthesise PK and PD data from the large and diverse body of literature on systemic CBD exposure in humans and animals. It comes with several strengths and limitations. The strength of the employed IB-de-risk approach is integration of all this data to make predictions of expected drug effects in humans. At the other side of the coin, effects of CBD were assessed on highly variable outcomes, including subjective, neurophysiological, autonomic and behavioural outcomes and changes in neuronal activity or connectivity were measured during various anxiety tests. Many of these tests elicit behaviour that belongs to an animal’s standard repertoire, and may not be controlled by the same neurobiological mechanisms as maladaptive avoidance behaviour in patients (Bach, 2022). Moreover, many anxiety tests are sensitive to specific classes of medication for anxiety, but less so to other drug classes (Griebel and Holmes, 2013). This may explain the inconsistent effects of CBD on anxiety outcomes and the absence of clear dose–effect patterns in the current work. A meta-analytic approach is needed to elucidate potential moderators of CBD effects, including type of anxiety test.

Some limitations are worth mentioning that are related to the imputations of missing PK data. First, the synthesis was limited to acute CBD effects. No PK estimates were made for multiple dose regimens, because this would require complex PK modelling and there was not sufficient data to reliably perform such calculations. PD drug effects may accumulate over time, or have a delayed onset (Agid et al., 2003). Depending on CBD’s mechanism of action for anxiety-reducing effects (the interested reader can refer to Crippa et al., 2018, for an overview), either an acute or (sub)chronic treatment regimen may be needed for the drug to reliably exert these effects.

Second, we accounted for type of formulation and diet in our imputations of missing PK data with oral administration of CBD in humans, because *C*_max_ (like *T*_max_) depends on the formulation used (Cherniakov et al., 2017: 27; Izgelov et al., 2020a: 3; Majimbi et al., 2021: 8). Furthermore, evidence exists that PK parameters in humans are affected by food intake (Taylor et al., 2018: 1064). For rats and mice, however, there was too little PK data looking into effects of different formulations and diets available to take these parameters into account. That being said, in rats and mice CBD was mostly administered via the i.p., instead of the oral route.

Unfortunately, however, only three studies reported PK data for i.p. administration (Deiana et al., 2012; Javadi-Paydar et al., 2019; Zieba et al., 2019), which may have introduced another source of bias. That is, by basing our estimations of missing PK parameters on such sparse data we may have influenced the sorting on *C*_max_ and AUC across species, from which, indeed, no interpretable pattern could be identified.

## Conclusion

This systematic analysis of the literature regarding anxiety-reducing properties of CBD is a first attempt to estimate its active and safe dose range in humans, from a translational cross-species perspective. The majority of effects were null effects, and anxiety-reducing effects were not concentrated in a particular range of blood levels across species, although some evidence for an inverted U-shaped dose–response curve was perhaps suggested when looking within species. So far, human studies that use oral doses in the 300–600 mg range tend to report anxiety-reducing effects. More data are needed to decide whether this range indeed provides a reliable anxiety-reducing effect, and what underlies the loss of a possible effect with higher concentrations seen in mice and rat studies.

## Recommendations for future work

The current systematic review yielded a mixture of beneficial and null effects of CBD on anxiety outcomes, which raises questions about the broad therapeutic use as a drug for anxiety. Meta-analyses may provide summary effects and investigate for which aspects of anxiety symptomatology CBD could be efficacious. A meta-analysis with this objective (PROSPERO CRD42021236572) is currently ongoing.

Furthermore, little is known about the pharmacological validity of preclinical anxiety tests for measuring the effects of CBD, which should include corresponding effects in preclinical anxiety tests and in humans who suffer from anxiety disorders (Ferreira et al., 2020). These knowledge gaps suggest fruitful avenues for future research.

In the current review, there was evidence of underreporting of aspects that could lead to bias in preclinical research, which included animal research and studies with PK and harm-related objectives. By reporting aspects of design, conduct, and analysis, confusion about underreporting or a study not possessing a certain quality (e.g. blinding) can be eliminated. Recommendations to optimising design, conduct and analysis of animal research are widely available (Vollert et al., 2020). The CONSORT extension on reporting of HARMS (Ioannidis et al., 2004) could be a useful guideline for studies with safety outcomes.

Lastly, there is an urgent need for integrated acute and (sub)chronic dosing PK/PD studies that measure both types of outcomes, especially in humans. This integration is needed to account for the influence of PKs on anxiety-reducing effects and to overcome the limitations inherent in synthesising these different types of data across publications and species. Together, these efforts will greatly advance the translation of preclinical research to clinical applications of CBD in humans.

## Supplemental Material

sj-docx-1-jop-10.1177_02698811221124792 – Supplemental material for Cannabidiol in clinical and preclinical anxiety research. A systematic review into concentration–effect relations using the IB-de-risk tool Click here for additional data file. Supplemental material, sj-docx-1-jop-10.1177_02698811221124792 for Cannabidiol in clinical and preclinical anxiety research. A systematic review into concentration–effect relations using the IB-de-risk tool by Caroline MB Kwee, Joop MA van Gerven, Fleur LP Bongaerts, Danielle C Cath, Gabriël Jacobs, Johanna MP Baas and Lucianne Groenink in Journal of Psychopharmacology

sj-docx-3-jop-10.1177_02698811221124792 – Supplemental material for Cannabidiol in clinical and preclinical anxiety research. A systematic review into concentration–effect relations using the IB-de-risk tool Click here for additional data file. Supplemental material, sj-docx-3-jop-10.1177_02698811221124792 for Cannabidiol in clinical and preclinical anxiety research. A systematic review into concentration–effect relations using the IB-de-risk tool by Caroline MB Kwee, Joop MA van Gerven, Fleur LP Bongaerts, Danielle C Cath, Gabriël Jacobs, Johanna MP Baas and Lucianne Groenink in Journal of Psychopharmacology

sj-docx-4-jop-10.1177_02698811221124792 – Supplemental material for Cannabidiol in clinical and preclinical anxiety research. A systematic review into concentration–effect relations using the IB-de-risk tool Click here for additional data file. Supplemental material, sj-docx-4-jop-10.1177_02698811221124792 for Cannabidiol in clinical and preclinical anxiety research. A systematic review into concentration–effect relations using the IB-de-risk tool by Caroline MB Kwee, Joop MA van Gerven, Fleur LP Bongaerts, Danielle C Cath, Gabriël Jacobs, Johanna MP Baas and Lucianne Groenink in Journal of Psychopharmacology

sj-jpg-2-jop-10.1177_02698811221124792 – Supplemental material for Cannabidiol in clinical and preclinical anxiety research. A systematic review into concentration–effect relations using the IB-de-risk tool Click here for additional data file. Supplemental material, sj-jpg-2-jop-10.1177_02698811221124792 for Cannabidiol in clinical and preclinical anxiety research. A systematic review into concentration–effect relations using the IB-de-risk tool by Caroline MB Kwee, Joop MA van Gerven, Fleur LP Bongaerts, Danielle C Cath, Gabriël Jacobs, Johanna MP Baas and Lucianne Groenink in Journal of Psychopharmacology

sj-xlsx-10-jop-10.1177_02698811221124792 – Supplemental material for Cannabidiol in clinical and preclinical anxiety research. A systematic review into concentration–effect relations using the IB-de-risk tool Click here for additional data file. Supplemental material, sj-xlsx-10-jop-10.1177_02698811221124792 for Cannabidiol in clinical and preclinical anxiety research. A systematic review into concentration–effect relations using the IB-de-risk tool by Caroline MB Kwee, Joop MA van Gerven, Fleur LP Bongaerts, Danielle C Cath, Gabriël Jacobs, Johanna MP Baas and Lucianne Groenink in Journal of Psychopharmacology

sj-xlsx-5-jop-10.1177_02698811221124792 – Supplemental material for Cannabidiol in clinical and preclinical anxiety research. A systematic review into concentration–effect relations using the IB-de-risk tool Click here for additional data file. Supplemental material, sj-xlsx-5-jop-10.1177_02698811221124792 for Cannabidiol in clinical and preclinical anxiety research. A systematic review into concentration–effect relations using the IB-de-risk tool by Caroline MB Kwee, Joop MA van Gerven, Fleur LP Bongaerts, Danielle C Cath, Gabriël Jacobs, Johanna MP Baas and Lucianne Groenink in Journal of Psychopharmacology

sj-xlsx-6-jop-10.1177_02698811221124792 – Supplemental material for Cannabidiol in clinical and preclinical anxiety research. A systematic review into concentration–effect relations using the IB-de-risk tool Click here for additional data file. Supplemental material, sj-xlsx-6-jop-10.1177_02698811221124792 for Cannabidiol in clinical and preclinical anxiety research. A systematic review into concentration–effect relations using the IB-de-risk tool by Caroline MB Kwee, Joop MA van Gerven, Fleur LP Bongaerts, Danielle C Cath, Gabriël Jacobs, Johanna MP Baas and Lucianne Groenink in Journal of Psychopharmacology

sj-xlsx-7-jop-10.1177_02698811221124792 – Supplemental material for Cannabidiol in clinical and preclinical anxiety research. A systematic review into concentration–effect relations using the IB-de-risk tool Click here for additional data file. Supplemental material, sj-xlsx-7-jop-10.1177_02698811221124792 for Cannabidiol in clinical and preclinical anxiety research. A systematic review into concentration–effect relations using the IB-de-risk tool by Caroline MB Kwee, Joop MA van Gerven, Fleur LP Bongaerts, Danielle C Cath, Gabriël Jacobs, Johanna MP Baas and Lucianne Groenink in Journal of Psychopharmacology

sj-xlsx-8-jop-10.1177_02698811221124792 – Supplemental material for Cannabidiol in clinical and preclinical anxiety research. A systematic review into concentration–effect relations using the IB-de-risk tool Click here for additional data file. Supplemental material, sj-xlsx-8-jop-10.1177_02698811221124792 for Cannabidiol in clinical and preclinical anxiety research. A systematic review into concentration–effect relations using the IB-de-risk tool by Caroline MB Kwee, Joop MA van Gerven, Fleur LP Bongaerts, Danielle C Cath, Gabriël Jacobs, Johanna MP Baas and Lucianne Groenink in Journal of Psychopharmacology

sj-xlsx-9-jop-10.1177_02698811221124792 – Supplemental material for Cannabidiol in clinical and preclinical anxiety research. A systematic review into concentration–effect relations using the IB-de-risk tool Click here for additional data file. Supplemental material, sj-xlsx-9-jop-10.1177_02698811221124792 for Cannabidiol in clinical and preclinical anxiety research. A systematic review into concentration–effect relations using the IB-de-risk tool by Caroline MB Kwee, Joop MA van Gerven, Fleur LP Bongaerts, Danielle C Cath, Gabriël Jacobs, Johanna MP Baas and Lucianne Groenink in Journal of Psychopharmacology
