# Supplementary material for: Cannabidiol in clinical and preclinical anxiety research. A systematic review into concentration–effect relations using the IB-de-risk tool
Source: J Psychopharmacol. 2022 Oct 14;36(12):1299–314. doi: 10.1177/02698811221124792 (PMC9716490; doi:10.1177/02698811221124792)
Supplement: sj-docx-1-jop-10.1177_02698811221124792 – Supplemental material for Cannabidiol in clinical and preclinical anxiety research. A systematic review into concentration–effect relations using the IB-de-risk tool [file sj-docx-1-jop-10.1177_02698811221124792.docx]

**Supplemental Material**

**Estimations of Cmax and AUC**

*Humans*

Given the study designs from the included studies with missing maximum plasma concentration (Cmax), intra-/extrapolations were based on studies that used a formulation to improve cannabidiol (CBD)’s bioavailability and tested subjects who were in a fed state. These studies are indicated by the grey data points in Supplemental Figure 1a, in which measured peak plasma concentrations (a) and area under the curve (b) are plotted against doses for oral CBD administration in human subjects.

For studies with missing area under the curve (AUC), intra-/extrapolations were based either on studies that used a formulation to improve CBD’s bioavailability and tested subjects who were in a fed state, or on studies with fasted individuals / studies that did not report a dissolving vehicle. This depended on the study designs of the studies with missing pharmacokinetic (PK) data.

Saturation of drug absorption appeared to occur at doses between 3000 and 6000 mg. Further, articles that focused on anxiety outcomes did not test CBD at such high dosages. Hence, we based our estimations of missing Cmax and AUC on PK data with CBD doses up to 1500 mg.

*a)*

*b)*

**Supplemental Figure 1.** Maximum plasma concentrations (a) and area under the curve (b) plotted against orally administered single CBD dose in humans. Two data points in the ‘fasted or naked’ condition are an approximation of true Cmax; in these studies too little measurements were taken to identify the peak concentration (Fusar-Poli et al., 2009, 2010; Verrico et al., 2020).

*Mice and rats*

With an eye on compatibility with studies with missing plasma concentrations, which were restricted to male animals, we only used data obtained from male animals for our predictions in mice (Deiana et al., 2012; Zieba et al., 2019) (Figure 3a) and in rats (Deiana et al., 2012; Javadi-Paydar et al., 2019) (Figure 3b). Of note, Javadi-Paydar et al. (2019) showed that in rats, bioavailability of CBD after i.p. administration was higher in females than in males. Estimations of Cmax after doses of 0.01-5 mg/kg i.p. in mice and 1-10 mg/kg in rats were negative. We used (fractions of) the lowest measured Cmax (Javadi-Paydar et al., 2019; Zieba et al., 2019) instead of intra-or extrapolation based on multiple data points to estimate these missing values. In Zieba et al. (2019) plasma levels were measured in the same mice who were subjected to behavioural tests, so in this case we used these PK data from the same article.

*a)*

*b)*

**Supplemental Figure 2.** Approximated maximum plasma concentrations plotted against single CBD doses administered intraperitoneally in male mice (a) and rats (b).Measured plasma concentrations with 15 and 30 mg/kg doses (Javadi-Paydar et al., 2019; Zieba et al., 2019) are approximations of true Cmax, too little measurements were taken to identify the peak concentration.

Two rodent articles had pharmacodynamic–pharmacokinetic objectives: One article with p.o. administration in mice (Deiana et al., 2012); and one with p.o. and subcutaneous administration in rats (Hložek et al., 2017). In the rat study, animals from the behavioural tests were reused for plasma level measurements more than 1 h after drug administration (Hložek et al., 2017).We used linear intra- or extrapolation for other studies with p.o. administration in mice and rats (see Supplemental Figure 3a and 4b). As in mice, the dose vs. approximated maximum plasma concentration relationship in rats was best described with a linear function (R^2^ = .64); an exponential function did not increase explained variance (R^2^ = .49).

*a)*

*b)*

**Supplemental Figure 3.** Maximum plasma concentrations plotted against single oral CBD dose in mice (**a)** and rats (**b)**.

Estimations of AUC after i.p. and oral administration in mice, and after i.p. administration in rats were based on PK data of Deiana et al. (2012), by taking fractions of their reported AUC values. Data on total systemic exposure from more than one article was available for oral administration in rats. We used linear interpolation to estimate missing AUC for this administration route and species. Extrapolation of AUC for a 10 mg/kg oral dose in rats (Hložek et al., 2017) yielded a negative value. We therefore used a fraction of the averaged lowest measured AUCs (Feng et al., 2021; Zgair et al., 2016) instead of linear interpolation to estimate this missing value.

**Supplemental Figure 4.** Area under the curve plotted against orally administered single CBD dose in rats.

**Amendments to the information provided in the protocol**

AUC was added as a second primary outcome next to Cmax.

Assessment of risk of bias for harm-related outcome was based on the CONSORT extension on reporting of HARMS (Ioannidis et al., 2004) rather than SYRCLE’s RoB (Hooijmans et al., 2014) or Cochrane RoB 2.0 (Sterne et al., 2019), because these tools are primarily aimed at efficacy outcomes.

**References**

Deiana S, Watanabe A, Yamasaki Y, et al. (2012) Plasma and brain pharmacokinetic profile of cannabidiol (CBD), cannabidivarine (CBDV), Δ9-tetrahydrocannabivarin (THCV) and cannabigerol (CBG) in rats and mice following oral and intraperitoneal administration and CBD action on obsessive-compulsive behaviour. *Psychopharmacology* 219(3): 859-873.

Feng W, Qin C, Chu Y, et al. (2021) Natural sesame oil is superior to pre-digested lipid formulations and purified triglycerides in promoting the intestinal lymphatic transport and systemic bioavailability of cannabidiol. *European Journal of Pharmaceutics and Biopharmaceutics 162: 43-49.*

Fusar-Poli P, Allen P, Bhattacharyya S, et al. (2010) Modulation of effective connectivity during emotional processing by Δ9-tetrahydrocannabinol and cannabidiol. *International Journal of Neuropsychopharmacology* 13(4): 421-432.

Fusar-Poli P, Crippa J, Bhattacharyya S, et al. (2009) Distinct effects of A9 Tetrahydrocannabinol and cannabidiol on neural activation during emotional processing. *Archives of General Psychiatry* 66(1): 95-105.

Hložek T, Uttl L, Kadeřábek L, et al. (2017) Pharmacokinetic and behavioural profile of THC, CBD, and THC+CBD combination AFTER pulmonary, oral, and subcutaneous administration in rats and confirmation of conversion in VIVO of CBD to THC. *European* *Neuropsychopharmacology* 27(12): 1223–1237.

Hooijmans CR, Rovers MM, de Vries RB, et al. (2014) SYRCLE’s risk of bias tool for animal studies. *BMC Medical Research Methodology* 14: 14-43.

Ioannidis JPA, Evans SJW, Gøtzsche PC, et al. (2004) Better reporting of harms in randomized trials: An extension of the CONSORT statement. *Annals of Internal Medicine* 141: 781-788.

Javadi-Paydar M, Creehan KM, Kerr TM, et al. (2019) Vapor inhalation of cannabidiol (CBD) in rats. *Pharmacology Biochemistry and Behavior* 184: 172741.

Sterne JAC, Savović J, Page MJ, et al. (2019) RoB 2: a revised tool for assessing risk of bias in randomised trials. *BMJ* 366: l4898.

Verrico CD, Wesson S, Konduri V, et al. (2020) A randomized, double-blind, placebo controlled study of daily cannabidiol for the treatment of canine osteoarthritis pain. *Pain* 161: 2191–2202.

Zgair A, Wong JC and Lee JB (2016) Dietary fats and pharmaceutical lipid excipients increase systemic exposure to orally administered cannabis and cannabis-based medicines. *American journal of translational research* 8(8): 3448.

Zieba J, Sinclair D, Sebree T, et al. (2019) Cannabidiol (CBD) reduces anxiety-related behavior in mice via an FMRP-independent mechanism. *Pharmacology Biochemistry and Behavior* 181: 93-100.
