# Supplementary figures and images for: Cannabidiol in clinical and preclinical anxiety research. A systematic review into concentration–effect relations using the IB-de-risk tool
Source: J Psychopharmacol. 2022 Oct 14;36(12):1299–314. doi: 10.1177/02698811221124792 (PMC9716490; doi:10.1177/02698811221124792)

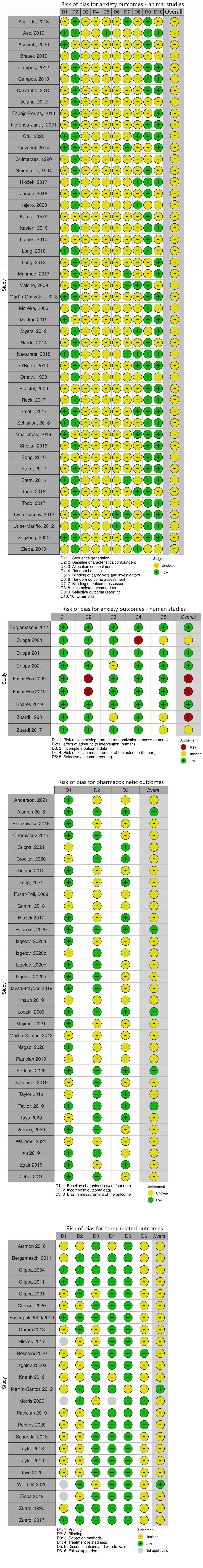

Supplement: sj-jpg-2-jop-10.1177_02698811221124792 – Supplemental material for Cannabidiol in clinical and preclinical anxiety research. A systematic review into concentration–effect relations using the IB-de-risk tool [file sj-jpg-2-jop-10.1177_02698811221124792.jpg]
